# Editorial: The cytoskeleton in T cell migration and activation

**DOI:** 10.3389/fimmu.2022.1057533

**Published:** 2022-10-14

**Authors:** Manish J. Butte, Jens V. Stein, Jérôme Delon

**Affiliations:** ^1^ Department of Pediatrics, David Geffen School of Medicine, University of California, Los Angeles, Los Angeles, CA, United States; ^2^ Department of Oncology, Microbiology and Immunology, University of Fribourg, Fribourg, Switzerland; ^3^ Université Paris Cité, Institut Cochin, Inserm, Centre National de la Recherche Scientifique (CNRS), Paris, France

**Keywords:** T cells, migration, activation, cytoskeleton, Rho (Rho GTPase), mechanical properties

Cytoskeletal elements and factors that regulate them are necessary for proper T cell-mediated immune responses. In this Research Topic, we gather reviews and original articles that emphasise the signalling pathways and mechanisms that regulate the T cell cytoskeleton during migration and activation.

One of the major signalling pathways that regulates the cytoskeleton are small GTPases of the Rho family (1). Although the roles of RhoA, Rac1 and Cdc42 in actin polymerization have been first reported in fibroblasts thirty years ago, the involvement of other less studied Rho members, including in immune cells, is less clear. In this Research Topic, Mokhtar et al. review the role of RhoG in T cells and provide evidence based on human patients devoid of RhoG function for a negative role of RhoG in T cell activation. Furthermore, Rho GTPases are activated by guanine nucleotide exchange factors (GEFs) such as DOCK2, which is the major Rac activator in T cells stimulated by the chemokine receptor CCR7. Here, Thelen et al. show that CCR7-driven intranodal T cell motility also involves to a minor extent the Tec kinase Itk downstream of PI3Kγ, whereas it does not require the Rac GEF Tiam1. Finally, the role of Rho GTPases in T cell biology extends far beyond their impact on actin polymerization, since they also control the activities of the actin cross-linkers ezrin/radixin/moesin (ERM) proteins. Moesin is the main member of this family expressed in T cells and a particular missense variant is responsible for a primary immunodeficiency disease in humans. In order to better understand the origin of this pathology, Avery et al. report here a mouse model of X-linked moesin-associated immunodeficiency (X-MAID) that shows severe defects in thymic T cell maturation and motility in response to sphingosine-1-phosphate but not in response to CCR7.

Two additional articles focus more specifically on proteins that increase actin polymerization downstream the LAT and SLP76 signalosomes triggered upon T-cell receptor engagement. First, Waldman et al. study Enabled/vasodilator-stimulated phosphoprotein (Ena/VASP) proteins which are a family of cytoskeletal effector proteins responsible for actin polymerization. Ena/VASP proteins contribute to T cell actin remodelling during T cell-APC interactions, which promotes the initiation of stable T cell conjugates during APC scanning. Therefore, Ena/VASP proteins are required for efficient activation and expansion of T cells *in vivo*. Second, Joshi and Morley summarise how the actin-bundling protein L-plastin (LPL) regulates T-cell activation and migration. LPL enhances F-actin polymerization and also directly binds to the β2 chain of the integrin LFA-1 to support intercellular adhesion and immunological synapse (IS) formation in human and murine T cells. T cells lacking LPL migrate slowly in response to chemoattractants such as CXCL12 and CCL19, and poorly polarise towards ICAM-1. Loss of LPL also impairs thymic egress and motility within lymph nodes. Thus, different actin modulators exhibit some degrees of redundancy to favour actin polymerisation and control the triggering of immune responses.

Furthermore, one particular property of T cells essential to fulfil their functions is their ability to polarise receptors, cytoskeletal components and organelles towards the antigen-presenting cell during assembly of the IS. In this Research Topic, Cassioli and Baldari review recent evidence regarding the presence of a puzzling network of actin filaments that surrounds the centrosome in resting T cells. Upon T-cell activation, centrosomal actin is depleted, allowing for centrosome detachment from the nucleus and its polarisation towards the immune synapse. This work reviews the clearance of actin by a centrosome-associated proteasome and the Bardet-Biedl syndrome 1 (BBS1) protein. In addition, González-Mancha et al. also report a role for the Sorting nexin 27 (SNX27) protein in centrosome and secretory machinery translocation to the immune synapse. In the absence of SNX27 expression, T cells show marked alteration in cytoskeleton architecture including a failure in the organisation of the microtubule network and defects in actin clearance at the IS. The possibility of a cooperation between BBS1 and SNX27 in order to regulate the same cytoskeletal phenomena remains elusive. Nevertheless, the polarity process that builds up during IS formation allows amplification and compartmentalization of signals in T-cell activation. As reviewed by Molon et al., the actin cytoskeleton together with CD28 and chemokine receptors play major roles in these events.

More recently, because the cytoskeleton can be considered as a soft material that confers cells a particular rigidity, physicists have been attracted towards this field and have thus embarked in studying the mechanical properties of immune cells using new tools and quantitative methods (2). In this Research Topic, Mustapha et al. introduce the method of Traction Force Microscopy for studying the forces that T cells apply onto their surrounding environment. For T cells to move within diverse tissues, blood and lymphatic vessels (3), which impose different types of physical constraints, T lymphocytes need to sense and adapt to their mechanical environments. T lymphocytes largely exert forces onto their environment through the LFA-1 integrin. Using optical tweezers, McDonald et al. report here the role of the LFA-1 partner kindlin-3 in its ability to regulate the adhesion of LFA-1 to ICAM-1. By measuring the force needed to dissociate a bead out of contact with T cells, the authors show that T cells bearing a pathogenic mutation of kindlin-3 show defective LFA-1-mediated T cell adhesion to the bead and weak, but not absent, catch bond formation. Thus signalling through kindlin-3 plays a role in catch bond formation and activation of LFA-1. These adhesive properties are most likely involved in the process of T cell scanning of their surrounding environment using their cell surface microvilli as sensors (4), a mechanosurveillance phenomenon that Göhring et al. review here.

Altogether, the articles gathered here bring additional information to the complexity of biochemical signals that control the T cell cytoskeleton. They also point to a more recent theme of research, which is the influence of mechanical signals on the cytoskeleton. The integration of biochemical and mechanical stimuli by the T cell cytoskeleton is an exciting field for further research in order to understand how morphology changes shape T cell migration and activation.

## Author contributions

JD wrote the first draft of the editorial. MB and JS edited the manuscript. All authors contributed to the article and approved the submitted version.

## Funding

JS is supported by the Swiss National Foundation project grants 31003A_172994 and 310030_200406, the Novartis foundation for medical-biological Research 21C171 and the San Salvatore Foundation. JD is supported by Inserm, Agence Nationale de la Recherche (ANR 2019 RIDES), Ligue contre le Cancer, and Idex Program Université Paris Cité.

## Conflict of interest

The authors declare that the research was conducted in the absence of any commercial or financial relationships that could be construed as a potential conflict of interest.

## Publisher’s note

All claims expressed in this article are solely those of the authors and do not necessarily represent those of their affiliated organizations, or those of the publisher, the editors and the reviewers. Any product that may be evaluated in this article, or claim that may be made by its manufacturer, is not guaranteed or endorsed by the publisher.
